# Epidemiology of ameloblastomas of the jaws; A report from the Netherlands

**DOI:** 10.4317/medoral.20316

**Published:** 2014-09-30

**Authors:** Marjolijn A E M. Oomens, Isaäc van der Waal

**Affiliations:** 1 Department of Oral and Maxillofacial Surgery/Pathology, VU University Medical Center (VUmc/Academic Centre for Dentistry (ACTA), Amsterdam, The Netherlands

## Abstract

Objectives: To provide epidemiological data of ameloblastomas of the jaws in the Netherlands over a 25-year time period (1985-2010) and to compare these data with data from other parts of the world. Material and Methods: The data of all patients diagnosed with a primary ameloblastoma of the jaws in the Netherlands in the period 1985-2010 have been retrieved from the nationwide histopathology and cytopathology network and registry in the Netherlands (PALGA). The pathology reports were screened and only those cases were included in which a distinct diagnosis of primary, histopathologically benign, intraosseous ameloblastoma was rendered. The average population in The Netherlands during this period amounted approximately 15 million people. Results: An annual incidence rate was approximately 1,5 per million population, the male-female ratio being 1.4: 1. The age at the time of diagnosis was 44.1 years. The average age in males was 46.3 years compared to an average age in females of 41.3 years, the difference being significant (p≤ 0.05). The results were compared with those available in only a small number of publications worldwide. Conclusions: There is no strong evidence for significant differences of the true incidence of ameloblastomas worldwide, neither for a gender predilection. The diagnosis is generally made at a somewhat lower age in women; this phenomenon is even much stronger in the Black population, irrespective of gender. No proper explanation for this finding can be provided.

** Key words:**Odontogenic tumor, ameloblastoma, epidemiology.

## Introduction

An ameloblastoma of the jaws is a neoplasm of odontogenic epithelial origin. Almost all ameloblastomas are histologically benign. Nevertheless, they may behave in a rather aggressive way by local recurrences when treated by enucleation only. In the World Health Organization classification of tumors, histologically benign ameloblastomas that metastasize are referred to as “metastasizing ameloblastoma”, while ameloblastomas that are histologically malignant, irrespective of the presence of metastases, are referred to as ameloblastic carcinomas (1).

Reported incidence figures of ameloblastomas are often derived from hospital pathology department records, being expressed as the relative incidence related to the total number of odontogenic tumors registered in that particular hospital (2-4). As described in a large review by Reichart *et al*., the average age of the reported patients at the time of the diagnosis varies in the various continents, being 42.3 years in Europe (5); in that review no figures could be provided for South America due a limited number of reported cases in the world literature. Remarkably, a significant difference was found between the average age and racial distribution, being 28.7 years in Blacks compared to 39.9 years in Caucasians.

The purpose of the present descriptive study is to provide epidemiological data of ameloblastomas of the jaws in the Netherlands over a 25-year time period and to compare these data with data from other parts of the world.

## Material and Methods

The data of all patients diagnosed with a primary ameloblastoma of the jaws in the Netherlands in the period 1985-2010 have been retrieved from PALGA, the nationwide network and registry of histopathology and cytopathology in the Netherlands (6). The pathology reports were screened and only those cases were included in which a distinct diagnosis of primary, histopathologically benign, intraosseous ameloblastoma was rendered. As a result, 591 cases were included. Of these cases, age and gender were recorded. The average population of the Netherlands in the study period amounted approximately 15 million, a large majority being of Caucasian background.

## Results

A total of 591 cases over a 25-year time period and a population of approximately 15 million in the Netherlands results in an annual incidence rate primary, histopathologically benign, intraosseous ameloblastoma of approximately 1,5 per million population. There were 341 males and 250 females, the male-female ratio being 1.4: 1. The age at the time of diagnosis was 44.1 years (range 0-98 years) with a peak incidence between the second and sixth decade. The average age in males was 46.3 years compared to an average age in females of 41.3 years, the difference being significant (*p*= 0.004).

## Discussion

In two reviews of the worldwide incidence of odontogenic tumors, including ameloblastomas, the relative frequency of these tumors as percentage of all odontogenic tumors has been determined, based on biopsy specimens submitted to departments of oral pathology (2,7). These figures, therefore, do not reflect the true incidence, being defined as “the number of new cases in a defined period of time in a defined population”. Only four studies could be retrieved from the English written literature in the past fourty years that were dealing with the true incidence of ameloblastomas, to be discussed hereafter.

In a Swedish ( Table 1) study of 49 cases of ameloblastoma in the period 1958-1971, reported to the Swedish Cancer Registry, histopathological reexamination resulted in exclusion of 18 cases (37%), 12 being other histopathologically benign lesions and 6 being malignancies (8). In addition, the Swedish authors corrected their findings because of suspected underreporting by clinicians or pathologists with a percentage of approximately 50. As a result, a true annual incidence figure of 0.60 per million population was established.

**Table 1:**
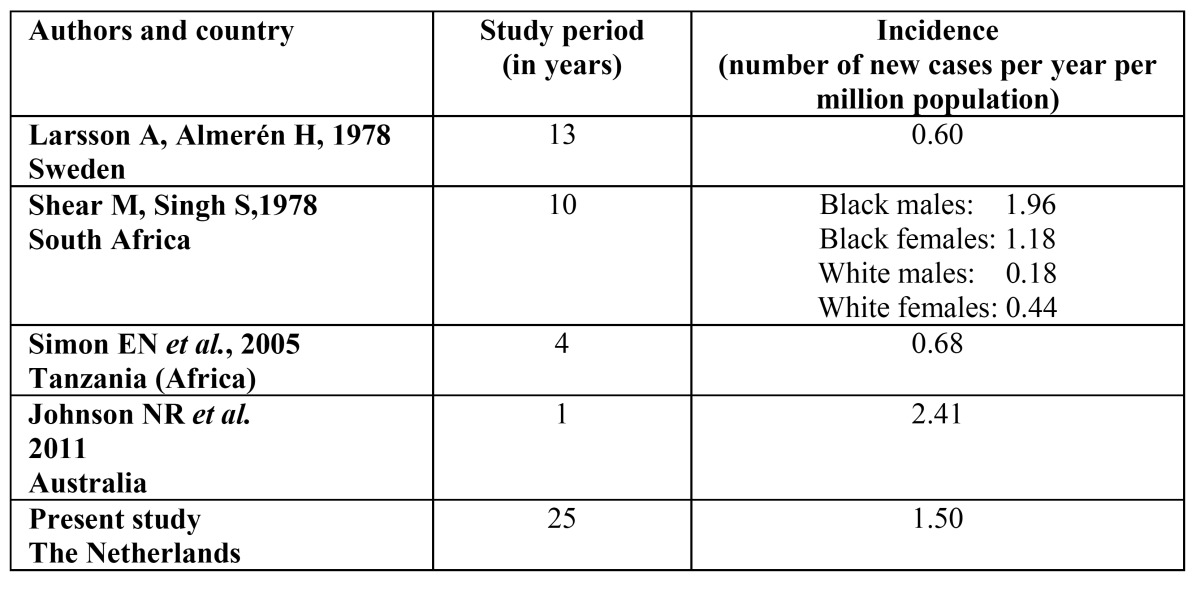
Incidence figures for ameloblastomas.

In the 10-year period 1965-1975 in which 42 cases of ameloblastoma were registered in Witwatersrand, South Africa, the annual incidence rates of ameloblastoma, standardized against the standard world population, for Black males, females and White males, females were 1.96, 1.20, 0.18 and 0.44 respectively (9). Apparently, no explanation could be provided for the low figures both in White males and females and the 1:2 male-female ratio in that study. In a 4-years prospective study in a Black African population in Tanzania in the period 1999-2003, an annual incidence rate of 0.68 was recorded (10). The authors concluded that their figure does not support the suggestion that ameloblastomas have a higher incidence in Black Africans than in other parts of the world, although they left room for the possibility that not all ameloblastomas in Tanzania are sent in for histopathological examination.

In a one year study in 2011 in Queensland, Australia, an incidence figure for ameloblastoma of 2.4 per million was recorded (11). Obviously, one should be careful to draw any firm conclusions from figures drawn from such a short period of time.

In the present study the data from the Netherlands in the period 1985-2010 have been collected retrospectively and have been retrieved from the nationwide histopathology network (PALGA). All pathologists in the Netherlands automatically register all their diagnoses, whether benign or malignant, to this Registry. Only in the period 1985-1991 a few percent of the pathologists had not yet reported all their diagnoses to the Registry. Based on the reports of the pathologists 132 (18%) out of the 723 cases have been excluded in this study. For practical reasons no attempt has been made to reexamine the histopathological specimens of the 591 included cases. Therefore, the annual incidence figure of approximately 1.5 per million population is perhaps a too high figure. On the other hand, there may have been cases of histopathological underdiagnosis of ameloblastomas.

In the present study a male-female ratio of 1.4: 1 was observed, being similar as in the Swedish study (8). In a large review of 2280 cases collected from the literature in the period 1960-1993 an almost equal male-female ratio of 1.14:1 was noticed (5). Also in a multicentric international study of 1289 cases in the period 1993-2009 an almost equal male-female ratio was observed (12).

The age at the time of diagnosis in the present study amounted 44.1 years with a peak incidence between the second and sixth decade. In a study from Brazil of 121 cases, the average age was 33.2 years (13). In the study by Reichart *et al*. the average age ranged from 30.4 years in Africa to 42.3 years in Europe (5). Ameloblastomas apparently are diagnosed at an earlier age in Africa than in other parts of the world ( Table 2).

**Table 2:**
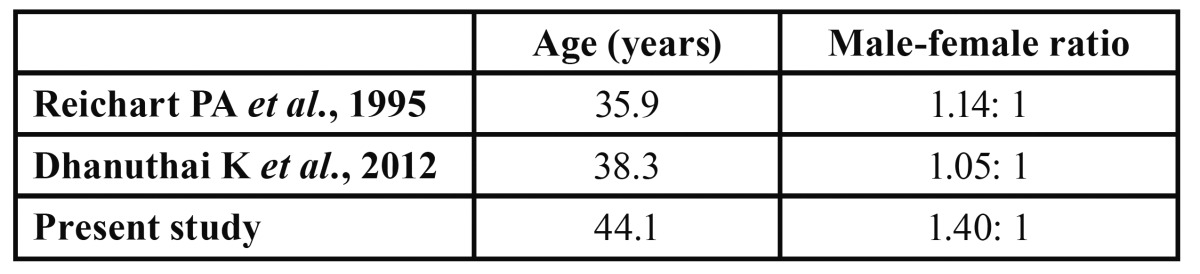
Age at the time of diagnosis and gender (m/f).

No proper explanation can be provided for this significant difference. Furthermore, both in the present study as in the large series reported by Reichart *et al*. a somewhat lower, but still significant (p≤ 0.05), average age of 4-5 years in females as compared to males was observed (5). No proper explanation can be provided for this difference either.
